# Treatment of an Askin tumor: A case report and review of the literature

**DOI:** 10.3892/ol.2013.1488

**Published:** 2013-07-24

**Authors:** XUE DOU, HONGJIANG YAN, RENBEN WANG

**Affiliations:** Department of Radiation Oncology, Shandong Cancer Hospital and Institute, Jinan, Shandong 250117, P.R. China

**Keywords:** Askin tumor, peripheral primitive neuro-ectodermal tumor, Ewing sarcoma family of tumors, treatment

## Abstract

Askin tumors are rare malignant neoplasms that are localized in the thoracopulmonary region and mainly occur in children and adolescents. Further investigation with regard to the effective treatment of this disease is required, since the disease has a low incidence and limited knowledge is available on the biological activity and prognostic factors of this type of tumor. The present study describes the case of a 30-year-old male patient with a histologically and morphologically proven Askin tumor who was treated in Shandong Cancer Hospital and Institute (Jinan, China). A chest computed tomography (CT) scan demonstrated a large mass filling the entire left lung, associated with mediastinum and right lung compression and accompanied by destruction of the 2nd rib. The patient accepted chemotherapy and radiotherapy instead of a radical mass resection since the mass was irresectable. A good clinical response was achieved to the chemotherapy and radiotherapy. The diagnosis and treatment of Askin tumors remains a challenge for clinicians and surgeons due to the absence of standard therapeutic guidelines for the treatment of this disease. According to the experience obtained from the cases encountered to date, treatment strategies should aim to reduce local recurrence and distant metastasis. Moreover, surgery, chemotherapy and radiotherapy or a combination of these methods appears to constitute an effective treatment strategy for Askin tumors.

## Introduction

The Ewing sarcoma family of tumors (ESFTs) is a group of rare malignancies arising from the migrating cells of the neural crest, characteristically composed of small cells arranged in cords and embedded in fibrous tissue. This group of malignancies includes classic Ewing sarcoma of the bone, extraskeletal Ewing sarcoma, peripheral primitive neuroectodermal tumors (PNETs) and Askin tumors. The tumors that occur in the thoracopulmonary region have been referred to as Askin tumors since the first report of a PNET of the chest wall by Askin *et al*(1) in 1979. The generally accepted view is that the neoplasms are essentially the same entity showing various degrees of neuroectodermal differentiation, and that Ewing sarcoma is considered to be the beginning of the Ewing sarcoma/PNET spectrum, while PNET is the end.

An Askin tumor is a soft tissue sarcoma belonging to the ESFTs that is localized in the thoracopulmonary region. This type of tumor mainly occurs in children and adolescents. Although rare, individual cases have been occasionally reported in older patients (2) and newborns (3). The incidence of Askin tumors is more frequent in males than in females (1.5:1) (4). This neoplasm usually arises from the soft tissue of the chest wall, although certain tumors have been reported to be localized in the lung (5). Pathologically, an Askin tumor is a malignant small round cell tumor that is known to be derived from neuroectodermal cells due to their cytogenetic appearance. The tumors show a common and unique chromosomal translocation, t(11;22)(q24;q12) (6). Based on the results following histological analysis, there are numerous overlapping clinical and pathological characteristics and therapeutic approaches between Askin tumors/PNETs and Ewing sarcoma. Written informed consent was obtained from the patient.

## Case report

**Figure 1:**
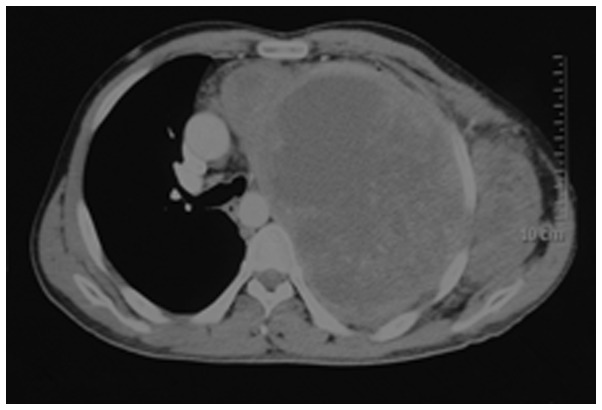
Axial computed tomography (CT) image showing a large mass infiltrating the chest wall with 2nd rib destruction.

**Figure 2:**
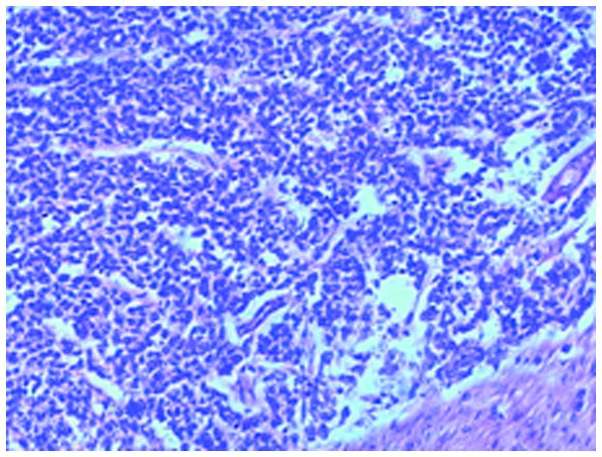
Histological analysis showing small round cells with dense nuclei and scant cytoplasm following observation under a light microscope (Hematoxylin and eosin; magnification, ×100).

A 30-year-old male was admitted to the People’s Hospital of Tai’an City in May 2012 presenting with a cough and intermittent vertigo. The patient was diagnosed with a pulmonary infection of the left lung and the condition improved with antibiotic treatment. Unfortunately, the same syndrome recurred 2 months later along with the presence of a swelling mass in the left chest wall and pain at the site of the swelling of the affected thoracopulmonary region. There was no history of fever, hemoptysis, dyspnea or joint pain. The patient was subsequently referred to the Shandong Cancer Hospital and Institute (Jinan, China) for further examination. A physical examination indicated swelling over the left chest wall and supraclavicular region, with decreased breath sounds over the left lung and dull percussion notes in the left hemithorax. No lymph nodes were palpable. Horner syndrome was also observed, as characterized by the symptoms of vertigo and left-sided anhidrosis and hypothermia without ptosis of the face and body, as well as miosis and conjunctival congestion in the left eye. Contrast-enhanced computed tomography (CT) showed a large, heterogeneous density compatible with areas of non-enhancing necrosis; the maximum cross-sectional area of the mass was 15×22.5 cm^2^. Compression of the mediastinum and right lung was evident. The large mass extended into the lung, chest wall and axilla, accompanied by destruction of the 2nd rib (Fig. 1). A needle biopsy was performed under radiological guidance, and a subsequent histological analysis showed that the tumor was composed of small round cells with scant cytoplasm and stained positive for neuron-specific enolase (NSE), CD99 and vimentin (Fig. 2). The diagnosis of an Askin tumor was confirmed following a morphological and immunohistochemical analysis.

The patient underwent chemoradiotherapy instead of surgery due to the anatomical complexities of the involved structures. The patient was administered 4 cycles of combined chemotherapy (1 cycle every 3 weeks), including 2 mg vincristine, 30 mg epirubicin and 60 mg cisplatin; the 4th cycle was administered in combination with radiotherapy at a dose of 1.8 Gy per fraction. The patient refused further treatment when 20 fractions of radiotherapy were completed. The patient response to the aforementioned treatment was good and a clinical response was achieved. A physical examination indicated disappearance of the swelling in the chest wall and relief of the symptoms, although a CT scan indicated that the limited reduction in tumor size was not ideal. At the time of writing this manuscript, the patient was well with no evidence of metastasis.

## Discussion

Askin tumors/PNETs are highly aggressive, and local recurrence and remote metastases are common. This type of neoplasm typically involves the periosteum, soft tissues and extrapulmonary tissue of the thoracic wall, but it may also involve peripheral lung tissue by direct local extension or occur in the primary peripheral lung cancer tissue (7). PNETs of the lung have been suggested to be more aggressive compared with PNETs in other locations (8). The metastatic sites include the lungs, the mediastinal and retroperitoneal lymph nodes, the extrathoracic skeleton, the liver, the adrenal glands and the sympathetic nerve chain (9). Askin *et al*(1) reported that 14/18 patients succumbed to the disease 4–44 months following the diagnosis and that the mean survival period was 8 months. Contesso *et al*(10) reported that the 2- and 6-year survival rates were 38 and 14%, respectively. After failure of local control, the mean survival rate was reported to be reduced to 11 months (11). The initial tumor mass (>100 ml), the histopathological response to initial chemotherapy and the presence of metastases at diagnosis were identified as major prognostic factors (12,13). An early diagnosis and multidisciplinary treatment modalities are considered to be significant factors for improving treatment outcomes.

The treatment of an Askin tumor should aim to control local disease and distant metastasis. Thus, the prevailing treatment of an Askin tumor is a combination of neoadjuvant chemotherapy, radical surgical resection and adjuvant chemotherapy and radiotherapy. Several studies have proved that this aggressive therapy may lead to a longer relapse-free survival (14,15). Consequently, in the present study, recent cases of Askin tumors and their treatment were reviewed. It was identified that almost all the patients underwent a multidisciplinary treatment including surgery, radiotherapy and chemotherapy.

### Surgery

The satisfactory outcomes that have been achieved to date may be attributed to comprehensive treatment. Surgery, as an option in the treatment of an Askin tumor, is crucial for local control due to the removal of the tumor itself, while chemotherapy/radiotherapy are administered as supplementary treatment. Numerous researchers have reported satisfactory results following surgery. According to a study on malignant chest wall tumors in children and young adults by Dang *et al*(16), surgical resection with en bloc removal of adjacent muscles or organs and chest wall reconstruction provided excellent local control of malignant chest wall tumors. According to a study conducted in the Memorial Sloan-Kettering Cancer Center (MSKCC) (17), complete remission was achieved in patients following surgery and chemotherapy rather than chemotherapy alone, thus underlining the importance of surgery. In this study (17), patients with large primary tumors that underwent surgical resection within 3 months of diagnosis were correlated with a significantly improved progression-free survival. Improved survival following surgical treatment has also been observed in other patient groups. Christiansen *et al*(14) described the cases of 8 patients with Askin tumors who underwent integrated treatment. Overall, 4/8 patients who underwent a complete resection were alive after a median follow-up time of 30 months, while the remaining 4 patients with extended disease and marginal surgery succumbed to the disease. Demir *et al*(18) showed that patients who underwent a complete resection exhibited a higher 5-year survival rate compared with patients who underwent an incomplete resection (56 vs. 25%; P=0.13). With regard to repeated relapse cases, a surgical resection combined with chemoradiotherapy resulted in satisfactory survival rates. The case of a 16-year-old male with an Askin tumor was described by Takanami *et al*(11). The patient underwent surgery for the excision of the tumor and subsequently underwent 6 excisions for local recurrence, with follow-up post-operative chemotherapy and radiotherapy for 7 years. This was the first case to be reported with long-term survival after repeated resections of local recurrences of an Askin tumor.

However, there are contradictory opinions with regard to the necessity and importance of surgery. Firstly, although the most effective treatment is surgery, a macroscopically complete resection does not guarantee absence of local recurrence. This means that early local recurrence and distant metastases may still be observed following complete resection and adjuvant therapy. Secondly, surgery combined with chemoradiotherapy is effective in the case of localized tumors. However, the role of surgery remains controversial for some cases of metastatic disease. Gunluoglu *et al*(19) showed that pre-operative therapy reduces the tumor size and facilitates surgery, while it does not decrease the probability of early local recurrence and distant metastasis. Incomplete surgical excision is not effective for the treatment of this type of tumor. Although surgery appears to be an effective treatment strategy, no effect has been observed on the disease-free period.

It is widely accepted that certain conditions are required prior to surgery, including a small initial volume of the tumor, a good response to systemic therapy, an operable location and sufficient resection margins. Otherwise, surgery should not be used as the only local therapy.

With regard to the implementation of the surgery, it should be aggressive with the aim of achieving good margins of resection, since a PNET has a high tendency to recur locally. According to a retrospective analysis of 42 patients, 10/12 patients with relapsed disease underwent an intralesional or marginal resection (20). Soyer *et al*(21) suggested that the general approach should be to perform a wide excision of all the involved structures, regardless of the tumor size. Concerning primary tumors of the ribs, all the involved ribs and their adjacent muscles and underlying pleura should be excised. However, not all tumors are suitable for complete resection since the tumor is usually extensive and is located in a difficult anatomical site. Therefore, adjuvant therapy is often administered to decrease the size of the tumor prior to resection, as well as to treat potential micrometastasis.

### Radiotherapy

The main role of radiotherapy is to achieve a satisfactory control of the primary disease; radiotherapy is also administered as adjuvant therapy prior to or following resection. Pre-operative radiotherapy is particularly suitable for patients with a poor clinical response to the initial chemotherapy or for patients in whom further tumor regression would allow the possibility of function-preserving surgery. In Cooperative Ewing’s Sarcoma Studies (CESS) 81 and 86 and European Intergroup Cooperative Ewing’s Sarcoma Study (EICESS) 92, radiotherapy was used in ~87% of patients as a pro- or post-operative adjuvant therapy or as radical radiotherapy for unresectable tumors (22,23). Based on these studies, Schuck *et al*(22) concluded that irradiation alone or post-operative irradiation as local therapy had satisfactory outcomes in local control and patient survival. Based on the protocol used in the CESS 81 study, radiotherapy was demonstrated to be similarly efficient to surgery for small lesions (lesion volume, <100 ml); however, a trend towards a better prognosis in surgically treated patients with large lesions >100 ml in volume was observed (12). The implementation of three dimensional conformal radiotherapy (3D-CRT) and intensity-modulated radiotherapy (IMRT), including meticulous delineation of planning target volumes (PTVs), treatment planning and accurate execution, result in the local failure rate reducing from an unacceptable 28.5% in the patients with PNET of the chest wall in the CESS 81 study to 8.6% in the CESS 86 study. The results from the Memorial Sloan-Kettering study demonstrated that radiotherapy was an effective modality for local control, particularly for patients without metastases (13). Although certain studies have performed radical resections, in multiple metastatic settings, chemotherapy combined with irradiation is the standard treatment in patients with macroscopic lesions (14,24,25). External-beam radiotherapy constitutes an alternative treatment strategy in patients whose pathological complete response rates are low, indicating a high risk of local relapse. Furthermore, certain studies have also suggested intraoperative radiotherapy to be an alternative treatment method in which satisfactory efficacy was observed in patients with cancer recurrence (26).

The standard radiotherapy approach, where the treatment program included exposure to 45–60 Gy plus intensive use of cyclophosphamide/doxorubicin, was established by Miser *et al*(27). According to this study, patients with PNETs of the chest wall received 45 Gy to the tumor with a surrounding 2 cm margin. Following treatment, no recurrence was observed in the radiation field, while pleural recurrence was observed in 3 patients. Schuck *et al*(22) suggested that in-field recurrence should be expected when doses of >50 Gy are used. Although appropriate radiation fields being provided, marginal relapses cannot be completely prevented. Currently, the dose for adjuvant radiotherapy is usually between 20 and 60 Gy. Radiotherapy is rarely used as the preferred treatment of a primary tumor, unless the tumor is anatomically unresectable, such as the case presented in the current study.

However, in view of the radiation-related chest wall deformities of growing bones and the neurodevelopmental complications, such as pulmonary fibrosis, when large volumes of the lung are in the radiation field, as well as the fear of the late second malignancy, the radiotherapy should be individualized, particularly in younger patients. Radiation to the heart also increases the possibility of cardiomyopathy induced by doxorubicin (28). However, the application of IMRT solved the problem and provided superior dose coverage of the PTV with the aim of keeping the dose to the organ at risk (OAR) as low as possible (29). Adjuvant radiotherapy is used when there is a high risk of recurrence, such as in a incomplete tumor resection, residual microscopic disease or tumor contamination during surgery. Currently, most patients undergoing definitive surgery following initial chemotherapy have an increased possibility of a successful complete resection of the tumor, and as a result, radiation to the chest is avoided.

### Chemotherapy

Chemotherapy is the first choice for the treatment of Ewing sarcoma, and the subsequent combination of surgery and radiation constitute the standard therapy (30). However, no standard therapy is available for the treatment of Askin tumors due to the rarity of this disease and the poor prognosis. Based on the grouping of these two diseases into the same WHO classification in 2002, the therapeutic guidelines for Ewing sarcoma may be useful in guiding the treatment of Askin tumors. In certain cases, extended surgery followed by post-operative chemotherapy and radiotherapy is the first choice of treatment (31). A number of researchers have reported the potential advantages of pre-operative chemotherapy with reference to the treatment of Ewing sarcoma (32,33). Veronesi *et al*(32) summarized the benefits of pre-operative chemotherapy, including the reduction in the risk of intraoperative tumor rupture and tumor cell dissemination, the increase in the probability of R0 resection, the enhanced probability of post-operative function preservation by a more conservative surgical approach, the decrease in any occult distant spread and the provision of a pathological and clinical evaluation of the response that favors the choice of the best post-operative regimen of chemotherapy and radiotherapy. Sawin *et al*(33) demonstrated that pre-operative chemotherapy resulted not only in a reduced tumor volume (from 7,054 to 911 cm^3^), but also in prolonged survival rates. Demir *et al*(18) reported that neoadjuvant chemotherapy significantly increased the complete resection rate (P=0.027) and that the 5-year survival rates of the patients with or without neoadjuvant therapy were 77 and 37%, respectively (P=0.22). Concerning older patients, radical surgery may be avoided when a complete response is achieved following neoadjuvant chemotherapy (34).

Askin tumors/PNETs are highly sensitive to chemotherapy. Due to the characteristic high recurrence rates and the high likelihood of metastases of this disease, systemic chemotherapy should be prompt even though the disease is organ-confined (8). Originally, chemotherapy for Ewing sarcoma consisted of 12 cycles of vincristine, actinomycin D, cyclophosphamide and adriamycin (ADM; VACA) in low-risk tumors (extremity tumors <100 cm^3^). In the case of patients with a high risk of recurrence (central tumors ≥100 cm^3^), the chemotherapeutic regimen used included vincristine, actinomycin D, ifosfamide (IFM) and ADM (VAIA) (35). Subsequently, similar to the EICESS 92 protocol for high-risk patients, the chemotherapy regime included 14 cycles of etoposide, vincristine, actinomycin D, IFM and ADM (EVAIA). The therapy was repeated every 3 weeks (1 cycle), while ADM was replaced with actinomycin D (36). Grier *et al*(37) reported that adding IFM and etoposide to the standard therapy (VACA) significantly improved the outcomes for patients with non-metastatic Ewing sarcoma, primitive neuroectodermal tumor of the bone or primitive sarcoma of the bone. However, according to Miser *et al*(38), the outcomes were disappointing in patients with metastatic disease.

The improvement of the treatment strategy for Ewing sarcoma and PNETs may be inspirational to the treatment of Askin tumors. Currently, the regimen includes multiple agents, including doxorubicin, vincristine and cyclophosphamide, alternating with IFM and etoposide (39). Additional effective chemotherapy regimens have also been reported. Askin *et al*(1) and Contesso *et al*(10) considered the commonly used chemotherapy regimen to be VAC, although a combination of DDP and 5-fluorouracil (5-FU) were also effective. We demonstrated that a chemotherapy regimen of a combination of vincristine, epirubicin and cisplatin was effective and well-tolerated by patients with less side-effects. Recently, Japanese researchers (40) reported satisfactory results when an adult with a PNET with multiple lung metastases was treated with the ADM and IFM (AI) regimen. After 7 cycles of chemotherapy, the size of the primary tumor and the multiple lung metastases decreased. Therefore, the authors suggested that the AI regimen was effective for PNET treatment.

The main side-effect of intensive chemotherapy is bone marrow suppression. In the event of sufficient bone marrow recovery, the next cycle of chemotherapy may be postponed, with the addition of granulocyte-colony stimulating factor (G-CSF). Autologous bone marrow transplantation has been suggested to be an effective rescue treatment, but this remains controversial. It has been demonstrated that the use of high-dose chemotherapy followed by either autologous bone marrow transplantation or peripheral stem cell rescue is valuable in the treatment of Ewing sarcoma (41). This potential benefit may be applied to PNET, particularly to patients with metastasis whose prognosis is usually poor. Young *et al*(42) reported the use of bone marrow transplantation after intensive chemoradiotherapy in patients with sarcomas of the chest wall. The preliminary results were satisfactory with a significant complete response rate and acceptable morbidity rates. In addition, cardiac toxicity with the use of anthracyclines should attract considerable attention during chemotherapy, particularly in children.

In conclusion, Askin tumors/PNETs develop in soft tissues and the disease is detectable only when the tumor is large. Thus, Askin tumors are mostly diagnosed at an advanced stage, which is one of the most important reasons for a poor prognosis. Moreover, due to the rarity of this disease, relevant studies are mostly small-scale, single-institution clinical trials. The diagnosis and treatment of Askin tumors/PNETs remains a challenge for clinicians and surgeons due to the absence of standard guidelines. Notably, a wide resection combined with polychemotherapy has been shown to result in satisfactory local control. The results of local control and decreased distant relapse in patients administered neoadjuvant chemotherapy followed by a complete resection are superior compared with the results in patients who undergo primary surgery followed by chemotherapy and/or radiotherapy. Consequently, large-scale randomized trials are required for the development of effective treatment strategies for Askin tumors.
